# Huge chest wall tumor detected incidentally owing to upper airway symptoms

**DOI:** 10.1111/ped.15313

**Published:** 2022-10-30

**Authors:** Shinsuke Fukui, Hisayuki Miyagi, Daisuke Ishii, Masatoshi Hirasawa, Yoshiki Hirano

**Affiliations:** ^1^ Department of Pediatrics Nayoro City General Hospital Nayoro Japan; ^2^ Division of Pediatric Surgery, Department of Surgery Asahikawa Medical University Asahikawa Japan

**Keywords:** inflammation, lipoblastoma, pediatrics, thoracic wall, tumor

The patient was a 1‐year‐old girl who had been healthy since birth and who had no remarkable clinical history in the prenatal or postnatal period. She was brought to a local physician owing to fever and cough as primary symptoms. Chest radiography revealed a well circumscribed area of reduced radiolucency in the right upper lung region (Fig. 1a), and because chest computed tomography suggested the presence of a very large convex lens‐like tumorous lesion measuring 80 × 41 × 72 mm (Fig. 1b,c), she was referred to our hospital. After additional examination by magnetic resonance imaging, surgical removal of the tumor was performed under a differential diagnosis, which included lipoma, lipoblastoma, teratoma, and liposarcoma. The lesion was approached under general anesthesia and differential lung ventilation in the left lateral decubitus position. The tumor had its base in the parietal pleura of the anterior chest wall near the second rib, and it had penetrated the parietal pleura and spread into the thoracic cavity. The lesion was resected sufficiently and without leaving the base unremoved or rupturing the capsule. The postoperative course was uneventful, and the patient was discharged on the fifth postoperative day. Histopathological findings indicated a diagnosis of lipoblastoma. Presently, 1 year after surgery, no recurrence has been observed; however, follow up is ongoing.

**Fig. 1:**
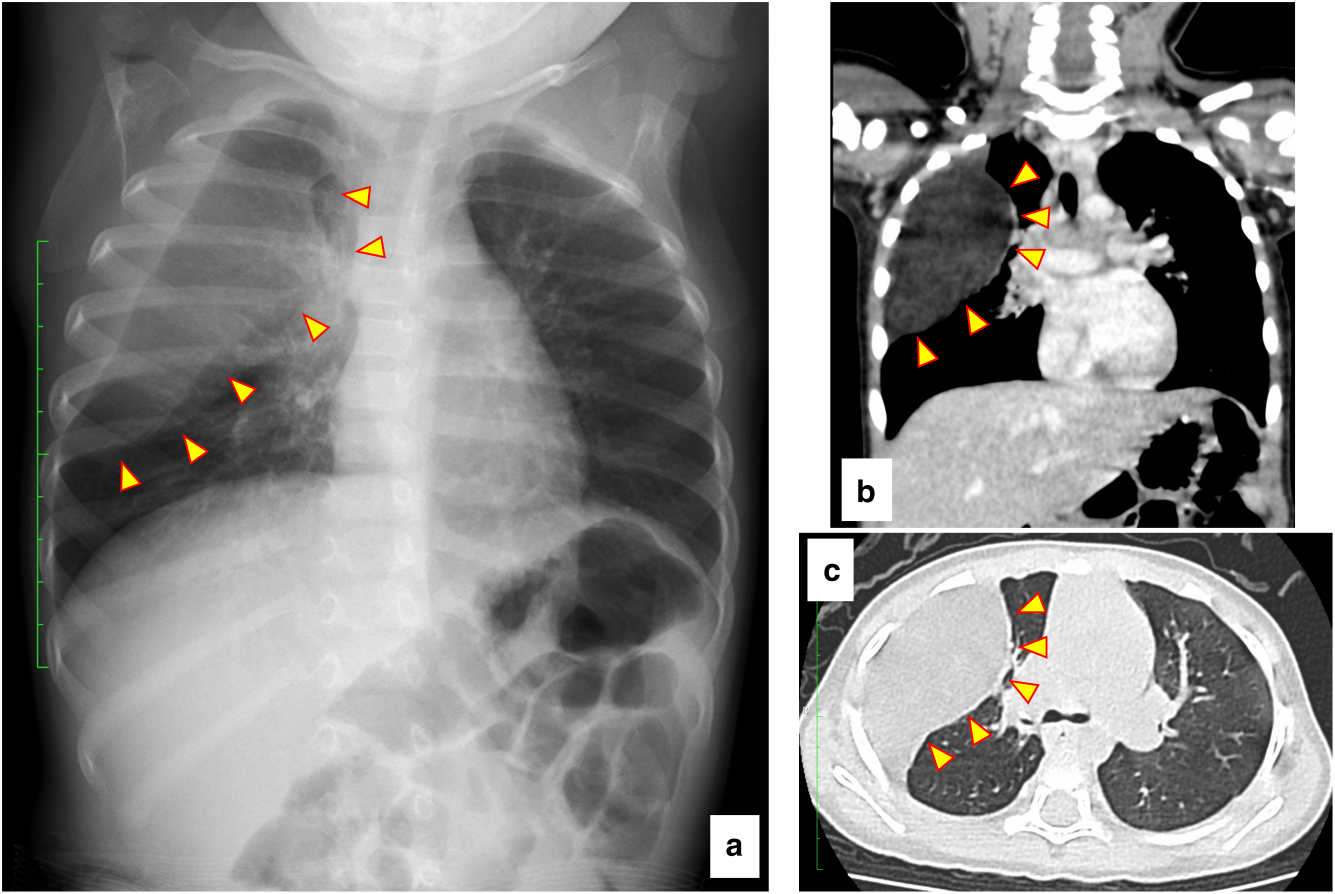
(a) Radiograph of the chest showing a convex lens‐shaped shadow extending from the right chest wall (yellow arrowheads). (b) Axial CT image: A tumorous lesion measuring 80 × 41 × 72 mm is visible along the chest wall in the right thoracic cavity (yellow arrowheads). (c) Coronal CT image: A convex lens‐shaped lesion is visible along the chest wall in the right thoracic cavity (yellow arrowheads). CT, computed tomography.

Lipoblastoma is a relatively rare benign mesenchymal tumor with a predilection for the limbs in children aged under 3 years.^1^, ^2^ Lipoblastoma may also develop in other areas, namely the head and neck, trunk, mediastinum, and retroperitoneum; however, reports of primary lipoblastoma of the chest wall are rare. The computed tomography and magnetic resonance imaging findings with lipoblastoma are not necessarily specific to this disease, and it is difficult to obtain a definitive diagnosis only by diagnostic imaging; therefore, the diagnosis should consider factors such as age.^3^ Primary lipoblastoma of the chest wall spreading into the thoracic cavity is difficult to detect without imaging examinations even if a patient is symptomatic. Primary lipoblastoma of the chest wall is considered extremely rare, with only 4.8% of the reported cases of lipoblastoma having arisen from this location; those spreading inside or outside the chest wall are even rarer, and patients often have no respiratory symptoms.^4^ Hicks *et al*. reported a recurrence rate of 14%–25% and that most recurrences were owing to incomplete resection.^5^ We therefore continue to follow our patient with semi‐annual ultrasonography.

In conclusion, a primary lipoblastoma of the chest wall is difficult to diagnose by visual or physical examinations, and it must be included in the differential diagnosis if a well‐circumscribed convex lens‐like lesion is detected by imaging examinations.

## Disclosure

The authors declare no conflict of interest.

## Author contributions

S.F. treated the patient and drafted the manuscript. H.M., D.I., and M.H. performed the surgical treatment and reviewed the manuscript. Y.H. reviewed the manuscript and provided conceptual advice. All authors read and approved the final manuscript.

## Informed consent

The patient's guardians provided written informed consent for the publication of this case report and accompanying images.
